# Single-cell transcriptomics combined with proteomics of intrathecal IgG reveal transcriptional heterogeneity of oligoclonal IgG-secreting cells in multiple sclerosis

**DOI:** 10.3389/fncel.2023.1189709

**Published:** 2023-06-08

**Authors:** Justyna Polak, Johanna H. Wagnerberger, Silje Bøen Torsetnes, Ida Lindeman, Rune A. Aa. Høglund, Frode Vartdal, Ludvig M. Sollid, Andreas Lossius

**Affiliations:** ^1^Department of Immunology, Oslo University Hospital, University of Oslo, Oslo, Norway; ^2^K.G. Jebsen Coeliac Disease Research Centre, University of Oslo, Oslo, Norway; ^3^Department of Molecular Medicine, Institute of Basic Medical Sciences, University of Oslo, Oslo, Norway; ^4^Department of Neurology, Akershus University Hospital, Lørenskog, Norway; ^5^Institute of Clinical Medicine, University of Oslo, Oslo, Norway

**Keywords:** multiple sclerosis, B cells, plasmablasts, oligoclonal bands (OCB), IgG, cerebrospinal fluid

## Abstract

The phenotypes of B lineage cells that produce oligoclonal IgG in multiple sclerosis have not been unequivocally determined. Here, we utilized single-cell RNA-seq data of intrathecal B lineage cells in combination with mass spectrometry of intrathecally synthesized IgG to identify its cellular source. We found that the intrathecally produced IgG matched a larger fraction of clonally expanded antibody-secreting cells compared to singletons. The IgG was traced back to two clonally related clusters of antibody-secreting cells, one comprising highly proliferating cells, and the other consisting of more differentiated cells expressing genes associated with immunoglobulin synthesis. These findings suggest some degree of heterogeneity among cells that produce oligoclonal IgG in multiple sclerosis.

## 1. Introduction

Multiple sclerosis (MS) is characterized by a persistent synthesis of IgG within the central nervous system (CNS). Accordingly, deposition of IgG and complement activation products are generally found in all active demyelinating lesions (Breij et al., 2008). In the cerebrospinal fluid (CSF), this locally produced IgG can be detected in more than 90% of the patients as oligoclonal IgG bands (Stangel et al., 2013), which is a diagnostic criterion for the disease (Thompson et al., 2018).

Although the role of oligoclonal IgG in MS is a subject of debate, increasing evidence supports the idea that it may contribute to the disease pathogenesis. Accordingly, the presence of oligoclonal IgG has been linked to higher levels of disease activity and disability, the conversion from a clinically isolated syndrome to definite MS, greater brain atrophy, and increased disease activity (Caroscio et al., 1986; Avasarala et al., 2001; Joseph et al., 2009; Ferreira et al., 2014; Heussinger et al., 2015; Farina et al., 2017; Seraji-Bozorgzad et al., 2017). Furthermore, a subset of recombinant antibodies constructed from clonally expanded antibody-secreting cells (ASCs) in MS CSF can cause complement-dependent cytotoxicity and demyelination in spinal cord explants and organotypic cerebellar slices (Blauth et al., 2015; Liu et al., 2017). Studies investigating the specificity of intrathecal ASCs have, however, revealed inconsistent results. Some studies have suggested reactivity against myelin-associated antigens (O’Connor et al., 2005; Kanter et al., 2006) and Epstein-Barr virus (Cepok et al., 2005; Lanz et al., 2022), but these findings are not consistent with those of independent studies (Owens et al., 2009; Sargsyan et al., 2010; Otto et al., 2011). Furthermore, one study suggested that some CSF IgG might be directed against intracellular autoantigens released during tissue destruction (Brändle et al., 2016).

The phenotypes of the B lineage cells that constitute the source of the oligoclonal IgG have not been settled, and to what extent these cells are susceptible to current immunomodulating strategies is controversial. Although a proportion of patients have been reported to lose detectable oligoclonal IgG after treatment with cladribine and natalizumab, most patients do seem to have a perpetuating intrathecal IgG synthesis despite highly effective immunomodulatory treatment (Cross et al., 2006; Harrer et al., 2013; Rejdak et al., 2019). This could indicate that the intrathecal IgG in these patients is synthesized by more differentiated long-lived ASCs within CNS survival niches (Eggers et al., 2017). Along the same line, it has been suggested that the development of secondary progressive disease in actively treated patients could be caused by therapy-resistant B lineage cells within such niches and that the presence of oligoclonal IgG might represent a useful endpoint for clinical trials (von Büdingen et al., 2017).

We previously used single-cell full-length RNA-seq and B-cell receptor reconstruction to analyze intrathecal B cells in MS (Lindeman et al., 2022). Here, we revisit the phenotype of the IgG-producing ASCs. To this end, we reanalyze the single-cell RNA-seq data from ten MS patients and combine this with mass spectrometry of intrathecally produced IgG.

## 2. Method

### 2.1. Patient inclusion and sample collection

The ten patients included in the study (Table 1) are part of a previously published cohort recruited at the Departments of Neurology at Akershus University Hospital and Oslo University Hospital, and details of sample acquisition and preparation are provided elsewhere (Lindeman et al., 2022). From this cohort, we chose patients who had a higher number of sorted and processed cells. MS9 and MS10 had previously been treated for 3 days with methylprednisolone; none of the other patients had received any type of immunomodulatory treatment at inclusion.

**TABLE 1:** Patient characteristics.

ID	Sex	Age	Diagnosis	CSF cell count^a^	OCB^b^	Albumin ratio	IgG index
MS1	M	36	SP-MS	36	+	12	1.7
MS2	F	26	RR-MS	4	+	3.8	1.1
MS3	M	20	RR-MS	19	+	6.2	0.81
MS4	F	46	RR-MS	19	+	3.1	0.72
MS5	F	31	RR-MS	33	+	3.2	2.1
MS6	M	52	RR-MS	6	+	5.0	1.1
MS7	F	44	RR-MS	18	+	6.4	1.1
MS8	M	21	RR-MS	21	+	6.4	0.95
MS9	F	48	RR-MS	17	+	4.3	1.3
MS10	M	35	RR-MS	5	+	3.3	0.75

^a^Number of mononuclear cells per microliter of CSF.

^b^More than two CSF-restricted oligoclonal bands on isoelectric focusing.

M, male; F, female; SP-MS, secondary progressive multiple sclerosis; RR-MS, relapsing-remitting multiple sclerosis.

### 2.2. Sample preparation and mass spectrometry

From each patient, we purified IgG from 1 ml of CSF and an equivalent amount of IgG from serum using Protein G Dynabeads (Thermo Fisher Scientific, Waltham, MA, USA). After elution in 20 mM hydrogen chloride, the buffer was exchanged to 50 mM ammonium bicarbonate. After reduction and alkylation (Høglund et al., 2019), 10 μg IgG in 12.5 μl buffer from each sample was transferred to new microcentrifuge tubes and 40 ng of trypsin (Promega, Madison, WI, USA) was added. After 45 min at 57°C in an orbital shaker, another 100 ng of trypsin was added, and the samples were further incubated for 90 min. The liquid chromatography mass-spectrometry analyses were performed in duplicates on a Q Exactive Orbitrap mass spectrometer equipped with an Easy nLC-1000 system (all from Thermo Fisher Scientific, Waltham, MA, USA) as previously described (Høglund et al., 2019).

### 2.3. Single-cell RNA-sequencing and processing of raw sequence data

The generation of the single-cell RNA-sequencing data set has been described before (Lindeman et al., 2022). In brief, we performed flow cytometry index sorting of CSF B lineage cells into 96-well plates (Bio-Rad, Hercules, CA, USA). Sequencing libraries were generated using an in-house modified Smart-Seq2 protocol and sequenced on an Illumina NextSeq500 platform (Picelli et al., 2014). After demultiplexing, the sequences were trimmed and filtered, and gene expression was quantified with Salmon version 0.11.3 (Patro et al., 2017). Quality control was done in R (R Core Team, 2022)/RStudio (Posit Team, 2022) with the scater package (McCarthy et al., 2017). BraCeR was used to reconstruct the full-length paired heavy- and light-immunoglobulin chains for each cell (Lindeman et al., 2018). Once the paired immunoglobulin sequences have been assigned to each cell, BraCeR groups productive immunoglobulin sequences for each locus in the cell population into clonal clusters using the “bygroup” subcommand of the Change-O toolkit’s “DefineClones” function (Gupta et al., 2015). Clonal grouping is based on the identification of common V- and J-gene sets among the sequences, equivalent CDR3 length, and CDR3 nucleotide distances < 0.2 as calculated using a human 5-mer targeting model (Yaari et al., 2013).

### 2.4. Analysis of mass spectrometry data

Mass spectrometry data was processed using MaxQuant v. 2.1.4.0 with the Andromeda search engine (Cox et al., 2014). Parameters of the search included label-free quantification and iBAQ values. The protein false discovery rate remained at 0.01, and methionine oxidation and acetylation of N-terminal amino acids were set as variable modifications. While running, multiplicates of the same sample match between runs feature was used, with minimum unique peptides set to 1. The library of sequences was constructed separately for each patient based on the amino acid translations of single cell RNA-seq full-length recombined V region of heavy- and light-chain immunoglobulin transcripts with an extension of 24 nucleotides into the constant region. The MaxQuant output was filtered for intrathecally produced IgG based on heavy/light-chain pairs with iBAQ_CSF_-iBAQ_serum_ > 50 k and iBAQ_CSF_/iBAQ_serum_ > 1.2, and single heavy chains meeting the same criteria with an additional requirement of at least three unique peptides identified.

### 2.5. Gene expression analyses and statistics

As outlined in our previous publication (Lindeman et al., 2022), we excluded immunoglobulin genes from the gene expression analysis prior to normalization. To minimize patient-to-patient variability and eliminate batch effects, we regressed out the number of detected genes and reads, percentage of mitochondrial genes, and patient-specific variation, while preserving the variability attributed to cell type. The gene expression analyses were performed in scanpy v.1.9.1 (Wolf et al., 2018), with the aid of scikit-learn v.1.0.2 for scaling the expression matrix (Pedregosa et al., 2011). For visualization, we used UMAP for dimension reduction (McInnes et al., 2018). The pathway enrichment analyses were performed in R/Rstudio using gprofiler2 v.0.2.1 (Kolberg et al., 2020), and visualized in Cytoscape v.3.9.1 (Shannon et al., 2003) using EnrichmentMap v.3.3 (Merico et al., 2010), ClusterMaker2 v.2.3.4 (Morris et al., 2011), and AutoAnnotate v.1.4 (Kucera et al., 2016). Additional figures were made in R/Rstudio with ggplot2 v.2 (Wickham, 2016), ggbreak v.0.1.1 (Xu et al., 2021), and ggpubr v.0.5 (Kassambara, 2023). We used two-sided non-parametric statistical tests with a significance level of 0.05.

## 3. Result

**FIGURE 1:**
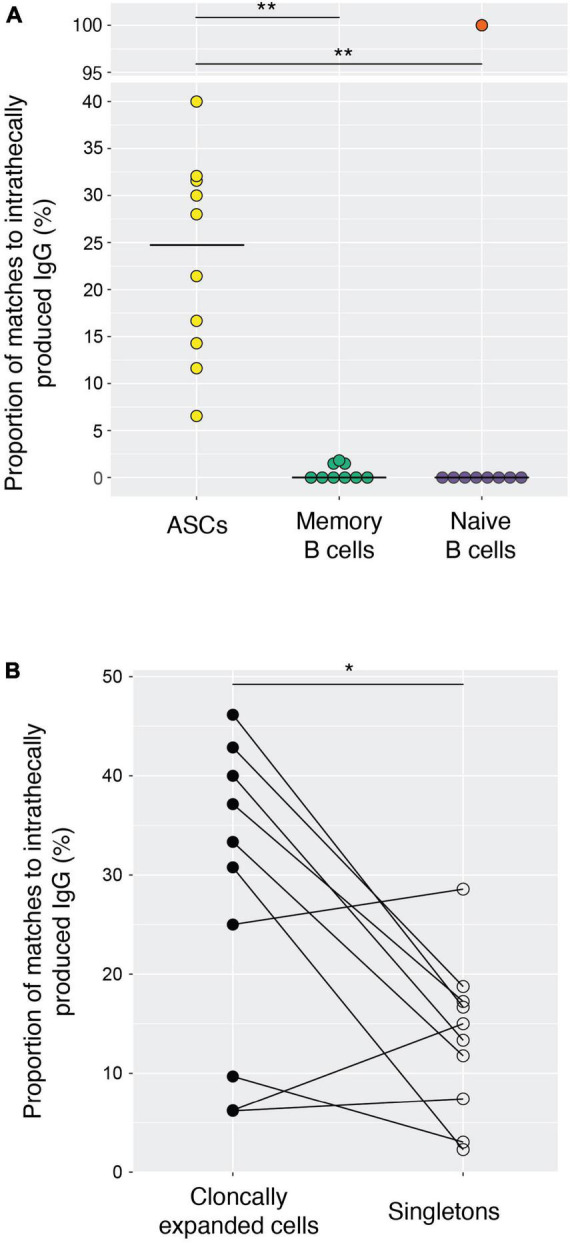
Intrathecally produced IgG matches clonally expanded antibody-secreting cells (ASCs) from the cerebrospinal fluid of MS patients. **(A)** The fraction of matches to intrathecal IgG for ASCs (642 cells), memory B cells (150 cells) and naive B cells (84 cells) was calculated for each patient, and each symbol indicates a patient. Clonally related cells were collapsed and treated as a single unit. Naïve and memory B cell populations were present in 9/10 patients. The outlier marked in red represents MS10, in whom we only analyzed a single sorted naïve B cell that also matched intrathecally produced IgG (excluded from statistical analysis). The horizontal lines depict the median values. Two-tailed Wilcoxon signed-rank test, **p < 0.01. **(B)** The proportion of clonally expanded ASCs vs. singletons matching intrathecally produced IgG. Each symbol indicates a patient (*n* = 10). Clonally related cells were collapsed and treated as a single unit. Two-tailed Wilcoxon signed-rank test, *p = 0.0371.

We analyzed IgG from CSF and serum from ten treatment-naive MS patients (Table 1) using mass spectrometry and combined this with single-cell RNA-seq data of sorted B lineage cells from the CSF (Lindeman et al., 2022). To determine the intrathecally synthesized IgG fraction and its cellular source, we performed label-free quantification of IgG in normalized CSF and serum samples matched to paired immunoglobulin heavy- and light-chain transcripts (Cox et al., 2014). We found that a median of 13.5% (range 3.6–32%) of all CSF B lineage cells (collapsed on a clonal level) matched intrathecally produced IgG. Reassuringly, we found that almost all these matches were found among ASCs (Figure 1A), whereas the number of hits were low among memory B cells and negligible in the naive B cell pool. The hits within the memory B cell population may be explained by the high degree of clonal relatedness between this population and the ASCs, as we have previously shown (Lindeman et al., 2022). The single-cell analysis data of B lineage cells allowed us to identify clonally expanded populations. A clonally expanded B lineage cell is here defined as a cell that has at least one other B lineage cell from the same patient with identical or related immunoglobulin heavy-chain sequences and identical or related light-chain sequences. The criteria for clonal grouping are outlined in the materials and methods. A singleton, on the other hand, is a cell that is not related in this way to any other sorted B lineage cell. Intrathecally produced IgG matched such clonally expanded populations more frequently than singletons (Figure 1B). Taken together, these results show that a proportion of clonally expanded ASCs sampled from the CSF faithfully represent ASCs that are producing oligoclonal IgG.

**FIGURE 2:**
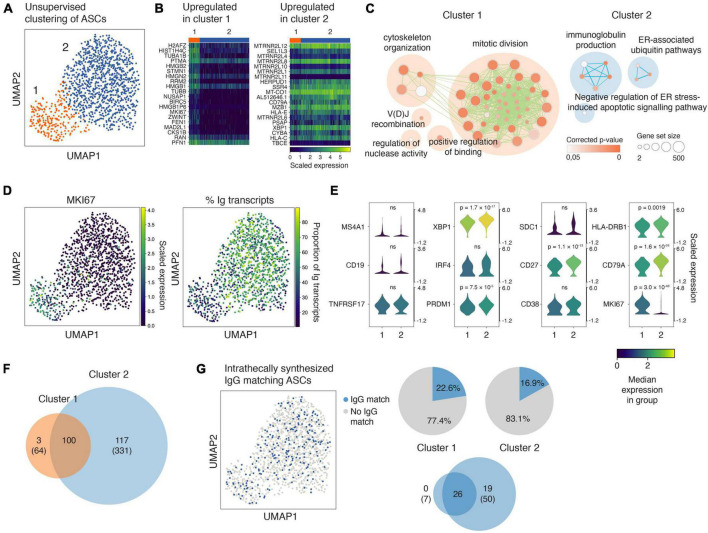
Antibody-secreting cells (ASCs) in the cerebrospinal fluid of MS patients cluster in two distinct yet clonally related groups, and intrathecally produced IgG matches cells from both groups. **(A)** UMAP projection and unsupervised clustering of intrathecal ASCs [*n* = 10 patients (1,283 cells)]. **(B)** Highly differentially expressed genes in each cluster compared to the other cluster. The genes are ranked according to the Mann–Whitney U test. **(C)** Enrichment map depicting Gene Ontology (GO) pathway enrichment analysis of highly differentially expressed genes in each cluster. Each node represents a GO biological process term. Related terms are clustered and labeled using the AutoAnnotate Cytoscape application following published protocols (Reimand et al., 2019). The lines (edges) between the nodes signify that genes are shared between the GO terms. **(D)** The expression of MKI67 and the proportion of immunoglobulin (Ig) transcripts superimposed on the UMAP projection of ASCs. **(E)** Violin plots showing the expression of genes of particular interest for ASCs in the two clusters. The genes in each cluster were compared in Scanpy using Wilcoxon rank-sum test, and the given *p*-values are adjusted for multiple testing using Bonferroni correction. ns: not significant. **(F)** Venn diagrams depicting the clonal overlap between ASCs in the two UMAP clusters. Given are the numbers of collapsed clonotypes and the number of singletons in brackets. The Venn diagrams are scaled according to the sum of collapsed clonotypes and singletons. Clonal relatedness was based on the identification of common V-and J-gene sets among the sequences, equivalent CDR3 length, and CDR3 nucleotide distances < 0.2 as calculated using a human 5-mer targeting model (Yaari et al., 2013). **(G)** UMAP projection of ASCs colored according to matches with intrathecally produced IgG. The pie charts show the proportion of ASCs matching intrathecally produced IgG in each UMAP cluster, and the Venn diagram shows the clonal overlap of these IgG-matching ASCs in each cluster (clonotypes collapsed and singletons in brackets).

The ASCs in the RNA-seq dataset are defined based on high surface expression of CD27 and CD38, and a proportion of immunoglobulin reads above 10% of the total transcriptome (Lindeman et al., 2022). We visualized the transcriptomes of the ASCs from the ten patients using UMAP and identified two distinct clusters (Figure 2A; Supplementary Figure 1). We identified the most differentially expressed genes between the clusters (Figure 2B) and performed a gene ontology (GO) enrichment analysis (Figure 2C). Whereas ASCs in the first cluster upregulated pathways involved in mitotic division and cytoskeleton organization, the ASCs in the other cluster upregulated pathways concerned with immunoglobulin synthesis and protein production (Figure 2C). Accordingly, the proliferation-associated gene *MKI67* was expressed by most cells in cluster 1, but to a much lesser extent in cluster 2 (Figure 2D). Further, the proportion of immunoglobulin transcripts per cell was larger in cluster 2 (Figure 2D), in which the cells also expressed somewhat higher levels of some plasma cell/plasmablast-related genes, including *XBP1* and *CD27* (Figure 2E). We observed strong clonal relatedness between the two clusters (Figure 2F; Supplementary Figure 2), which indicates a common origin and/or that they are in different stages of a maturation pathway. Oligoclonal IgG matched a comparable proportion of ASCs in each cluster (Figure 2G), and the majority of the IgG matched to ASCs that were clonally expanded across the clusters (Figure 2G, lower Venn diagram). Taken together, these data provide evidence of transcriptional heterogeneity of clonally related ASCs producing oligoclonal IgG in MS.

## 4. Discussion

In the present study, we used single-cell RNA-seq data and combined it with mass spectrometry to trace the oligoclonal IgG-producing cells. The results show that oligoclonal IgG matches with the transcriptome of both heavily proliferating ASCs and more differentiated ASCs that are mainly occupied with immunoglobulin production. The clonal relatedness between these populations with slightly different transcriptional profiles indicates a shared ancestry and/or that they are part of a developmental progression from newly generated plasmablasts to more differentiated phenotypes.

In a previous study, it was demonstrated that intrathecally produced IgG match B cell transcripts from the CSF, suggesting that oligoclonal IgG is secreted by ASCs that are present in the CSF (Obermeier et al., 2008). Nonetheless, several observations indicate that only a proportion of intrathecal ASCs are involved in the production of the main fractions of oligoclonal IgG. First, as demonstrated previously by us and others (Colombo et al., 2003; Greenfield et al., 2019; Tomescu-Baciu et al., 2019), there is a limited clonal overlap in CSF cell samples collected at different time points whereas the pattern of oligoclonal IgG is remarkably stable over time (Walsh and Tourtellotte, 1986; Axelsson et al., 2013; Tomescu-Baciu et al., 2019). This underscores that a finite sample of CSF cells only represents a small proportion of all ASCs in the CNS, and that any random sample will miss relevant oligoclonal IgG-secreting cells and include irrelevant B cell clones. Second, it is well-known that patients with MS have an intrathecal synthesis of IgG against disease-irrelevant pathogens, including measles, rubella and varicella zoster virus, which are not part of the major fractions of oligoclonal IgG (Vartdal et al., 1980; Feki et al., 2018). Therefore, pinpointing the disease-relevant ASCs that are responsible for the production of oligoclonal IgG might be key to dissect the mechanisms driving the intrathecal B cell response in MS.

Our study has several limitations. In order to accurately match oligoclonal IgG to specific subpopulations of B cells and perform a precise characterization and comparison of them, we selected patients from our previously published cohort who had a higher number of sorted and processed B lineage cells. Although this approach provided us with a larger amount of data for analysis, it is important to acknowledge that it might have introduced a selection bias, as it may have preferentially included patients with higher CSF cell numbers and potentially greater disease activity. Therefore, our findings may not be applicable to patients with less active disease. Furthermore, although we observed a lower expression of the proliferation marker *MKI67* in cluster 2, we cannot definitively determine whether these cells are more differentiated plasma cells or if they represent plasmablasts in the G1 phase of the cell cycle – transiently downregulating *MKI67*. Finally, the selected patients had different ages, and one of them had developed secondary progressive disease, indicating a longer disease duration. This heterogeneity may have introduced variability in our study.

Matching mass spectrometry data of IgG to immunoglobulin transcripts can be challenging due to the high degree of sequence homology between the transcripts (Snapkov et al., 2022). Here, we addressed this issue by setting strict criteria for what is considered a hit, requiring peptide hits for both the heavy and light chains of a given IgG molecule, or at least three hits for the heavy chain. However, these strict criteria may lead to a loss of sensitivity, and therefore our study might underestimate the true overlap between the immunoglobulin proteome and transcriptome in the CSF of MS patients. Another potential source of bias in our study is the intrathecal fraction of IgG that originates from serum, as less than 50% of intrathecal IgG in MS represents intrathecally produced oligoclonal IgG (Reiber et al., 1998). To account for this, we utilized label-free quantification of heavy and light chains in serum and CSF, measured by MaxQuant’s intensity-based absolute quantification (iBAQ) values, a measure of protein abundance (Schwanhäusser et al., 2011). Of note, all included mass spectrometry hits had an estimated abundance in the CSF of at least twice that in serum.

In conclusion, our study sheds light on the cellular origins of oligoclonal IgG in MS by combining single-cell RNA-seq data with mass spectrometry. Our findings suggest that oligoclonal IgG is produced by both heavily proliferating ASCs and more differentiated ASCs mainly focused on immunoglobulin production, which likely share a common ancestry or developmental progression. Overall, our study contributes to understanding the intrathecal B cell response in MS and highlights the need for further investigation to pinpoint the disease-relevant ASCs responsible for the production of oligoclonal IgG.

## Data availability statement

The datasets presented in this study can be found in online repositories. The names of the repository/repositories and accession number(s) can be found below: https://ega-archive.org, EGAS00001005745 and https://massive.ucsd.edu, MSV000091526.

## Ethics statement

The studies involving human participants were reviewed and approved by the Regional Ethical Committee South East, Norway (2009/23). The patients/participants provided their written informed consent to participate in this study.

## Author contributions

JP, AL, ST, LS, and FV contributed to the study design. JP, AL, IL, RH, JW, and ST contributed to the collecting and analyzing data. JP, JW, and AL drafted the manuscript. All authors contributed to revising the manuscript and approved the submitted version.
