# Supplementary material for: Single-cell transcriptomics combined with proteomics of intrathecal IgG reveal transcriptional heterogeneity of oligoclonal IgG-secreting cells in multiple sclerosis
Source: Front Cell Neurosci. 2023 Jun 8;17:1189709. doi: 10.3389/fncel.2023.1189709 (PMC10285169; doi:10.3389/fncel.2023.1189709)
Supplement: Supplementary file 1 [file Data_Sheet_1.PDF]

Supplementary Material

Single-cell transcriptomics combined with proteomics of intrathecal IgG reveal transcriptional heterogeneity of oligoclonal IgG-secreting cells in multiple sclerosis

Justyna Polak, Johanna H. Wagnerberger, Silje Bøen Torsetnes, Ida Lindeman, Rune A. Aa. Høglund, Frode Vartdal, Ludvig M. Sollid, Andreas Lossius*

*** Correspondence:** Andreas Lossius: andreas.lossius@medisin.uio.no

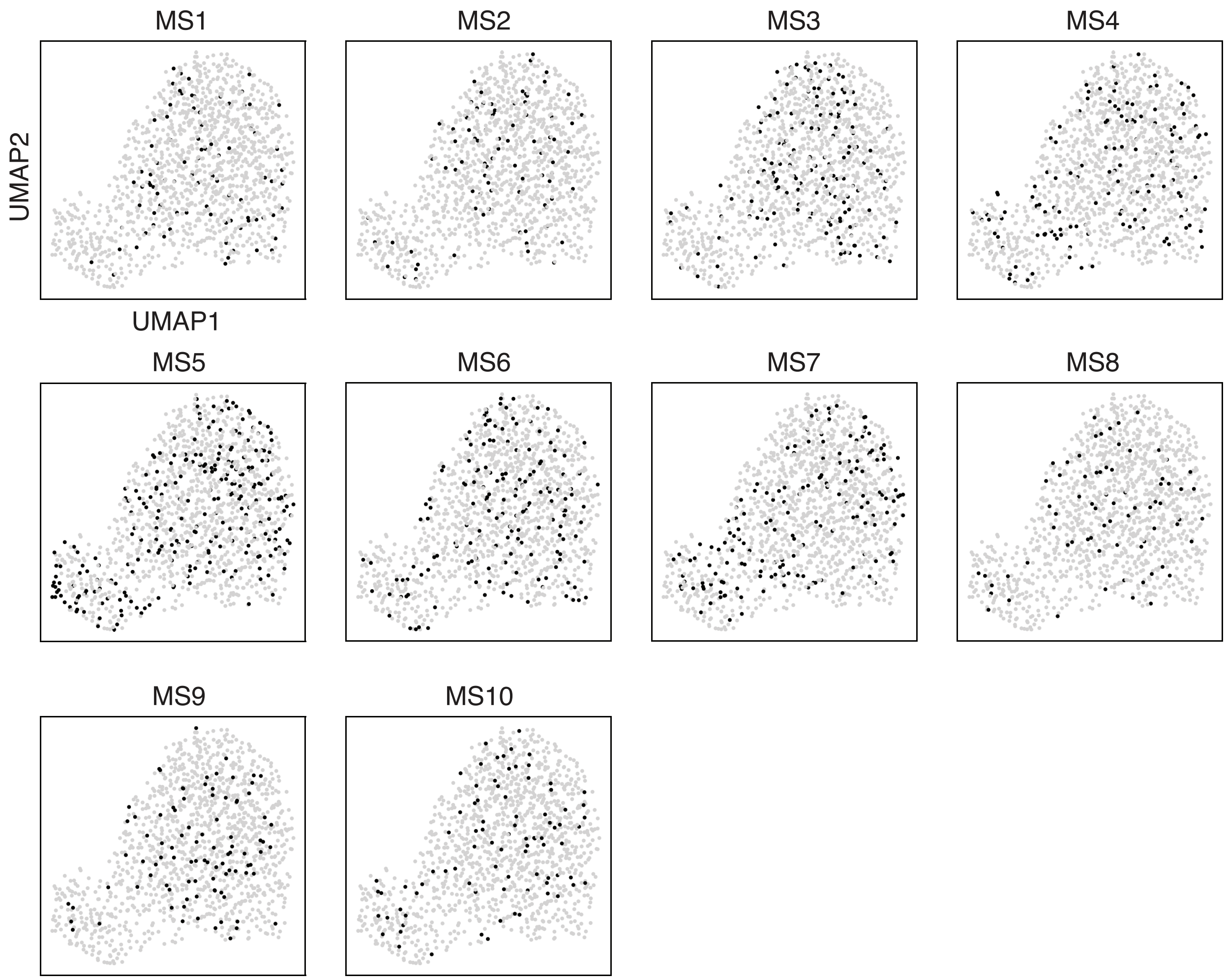

Supplementary Figure 1. UMAP projections of 1283 intrathecal antibody-secreting cells as in Figure 2A, D, and G according to individual patients.

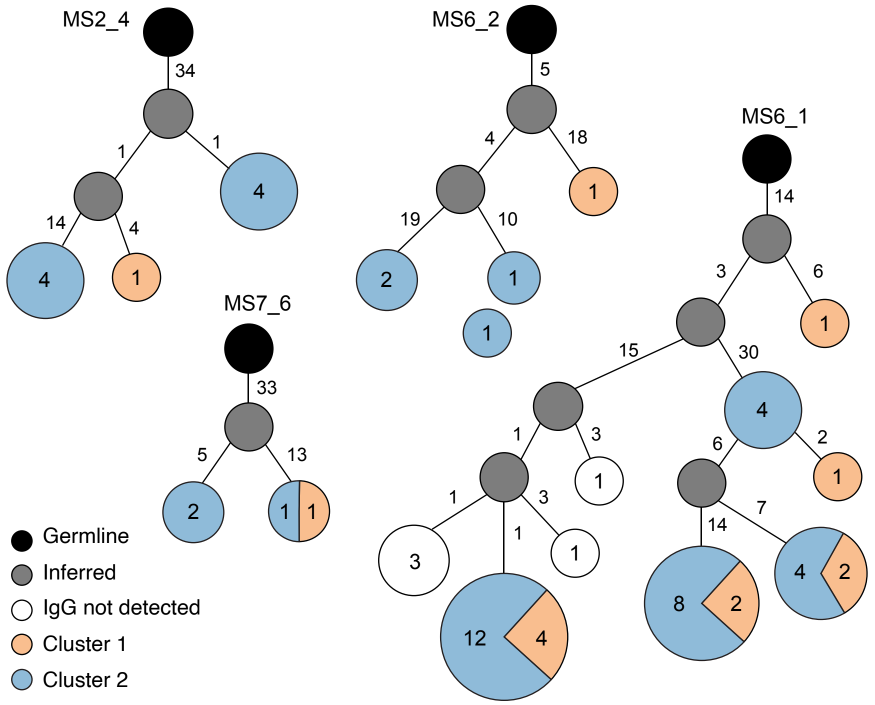

Supplementary Figure 2. Clonal connections across UMAP cluster 1 and 2 as shown in Figure 2A. Representative lineage trees of antibody-secreting cells with clonal members in both UMAP clusters with inferred germinal sequences at the root are shown. The size of the nodes and the number(s) inside the nodes reflects the number of cells with a unique B cell receptor sequence. The number of mutations between the nodes are shown next to each branch.
